# Extreme Genu Recurvatum Deformity in a Pediatric Patient With Spondyloepiphyseal Dysplasia: Gradual Correction With Z-plates and Hexapod Frame

**DOI:** 10.7759/cureus.25265

**Published:** 2022-05-23

**Authors:** James Seymour, Neeraj Vij, Mohan Belthur

**Affiliations:** 1 Orthopedic Surgery, MountainView Regional Medical Center, Las Cruces, USA; 2 Orthopedic Surgery, University of Arizona College of Medicine - Phoenix, Phoenix, USA; 3 Pediatric Orthopedics, Phoenix Children's Hospital, University of Arizona College of Medicine - Phoenix, Phoenix, USA

**Keywords:** epimetaphyseal dysplasia, ilizarov principles, pediatric orthopedics, knee deformities, skeletal dysplasias

## Abstract

Spondyloepiphyseal dysplasia is a type II collagenopathy with resulting spinal and extremity deformities. The clinical manifestations include short stature, hearing loss, kyphoscoliosis, and complex knee deformities. Genu recurvatum can be a challenging surgical problem, especially when the deformity is severe. In this report, we present a case of severe genu recurvatum in a 14-year-old female that was treated with a pediatric circular fixator with the addition of two z-plates. At one year follow-up, the patient demonstrated improved knee range of motion, tibial alignment with the radiographic union, and good ambulatory ability. The hexapod fixator with the use of two Z-plates may help ensure that appropriate ring strut angles are achieved. Larger studies regarding the efficacy of this treatment option in spondyloepiphyseal dysplasia are required.

## Introduction

Spondyloepiphyseal dysplasia congenita (SEDC) is a type II collagen synthesis disorder with resulting growth abnormalities in the spine and proximal epiphyseal centers. It is traditionally thought to result from a Collagen Type II Alpha 1 Chain (COL2A1) mutation on chromosome 12q13.1-q13.2 [1,2]; however, recently the Trafficking Protein Particle Complex Subunit 2 (TRAPPC2) gene mutation has also been associated with the skeletal dysplasia [3,4]. Salient features include short stature, flattened facies, myopia, retinal detachment, and hearing loss [5]. Musculoskeletal findings include atlantoaxial instability, thoracic kyphoscoliosis, flattened vertebral bodies, hip dysplasia, coxa vara, genu valgum, osteoporosis [6-8], and early-onset severe osteoarthritis [9,10].

Orthopedic treatment begins with conservative options including spinal bracing, physical therapy, and occupational therapy. Surgical management in children with spondyloepiphyseal dysplasia revolves around hip correction with valgus hip osteotomies [8] and spinal correction including atlantoaxial fusion [11] and posterior spinal fusion surgeries [12]. Corrective osteotomies for lower leg deformities have also been described.

The most common lower extremity deformity in SEDC is genu valgum. Genu recurvatum is a less commonly reported deformity. The correction of genu recurvatum has been well described with the use of the Ilizarov technique. However, the Ilizarov technique is not without its complications and several modifications have been proposed. In this report, we present a case of genu recurvatum in a patient with SEDC who was treated with gradual correction with a hexapod fixator augmented with two z-plates to facilitate easy correction as compared to a standard Taylor spatial frame (TSF).

## Case presentation

Statement of informed consent

The patient and her family were informed that the case would be submitted for publication. They verbalized understanding and provided their consent to do so.

History

We present the case of a 14-year-old female with a history of spondyloepimetaphyseal dysplasia who presents with bilateral extreme genu recurvatum left worse than right. The patient had a past surgical history of juvenile-onset scoliosis treated with growing rods, coxa vara treated with valgus proximal femoral osteotomies. She reported the associated symptoms of multidirectional knee instability, functional limitations, and undue stress to her torso and back.

Physical examination

On physical exam, the patient demonstrated a disproportionate short trunk status with dysmorphic features, well preserved spinal correction, and well-preserved hip correction with a good range of motion bilaterally. Physical examination of the knees demonstrated a left recurvatum deformity of 90 degrees, a right recurvatum deformity of 60 degrees, and no frontal plane malalignment. On clinical gait assessment, the patient could ambulate unassisted; however, this was difficult for the patient. A notable lateral trunk lean and out-toeing with external foot progression angles of -40 and -25 for the left and right, respectively, could be noted.

Imaging

**Figure 1:**
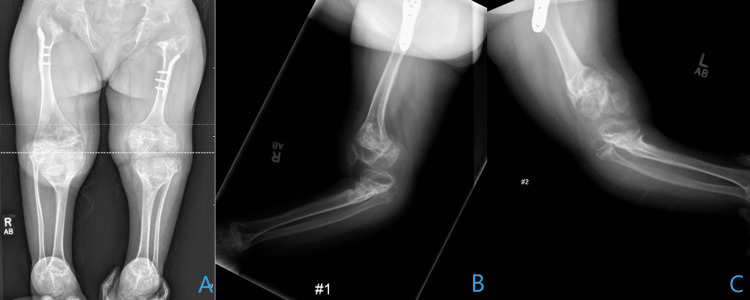
Radiography on initial presentation Anteroposterior (A) and lateral (B, C) radiographs of the lower extremity demonstrate a significant genu recurvatum bilaterally with contributions largely from the tibia on the right (B) and contributions of about two-thirds from the tibia and one-third from the distal femur on the left (C).

Radiography demonstrated bilateral genu recurvatum deformity that was predominantly due to a proximal tibial recurvatum deformity (Figures 1A, 1B).

Treatment

**Figure 2:**
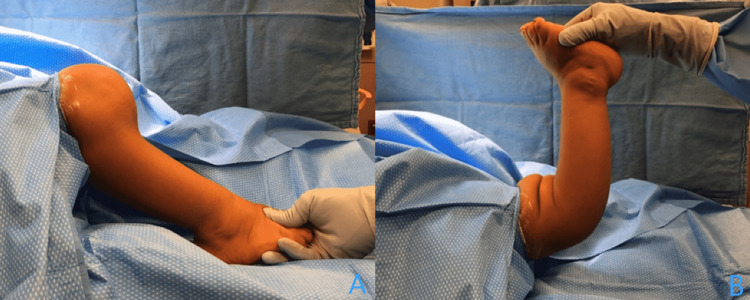
Operating room examination Examination in the operating room demonstrate the severity of the deformity in both flexion (A) and extension (B).

**Figure 3:**
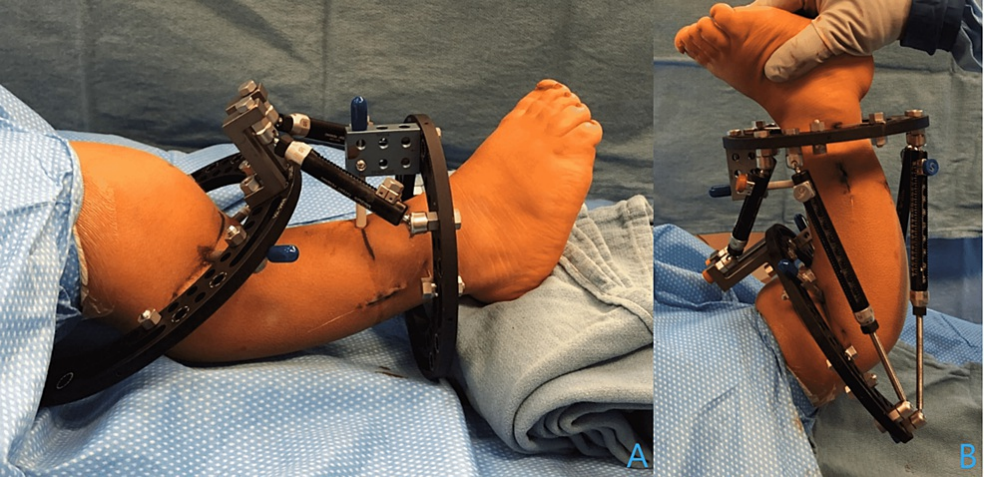
Placement of circular external fixator Clinical pictures after placement of the circular external fixator demonstrate no block of previous motion in neutral (A) and extension (B).

**Figure 4:**
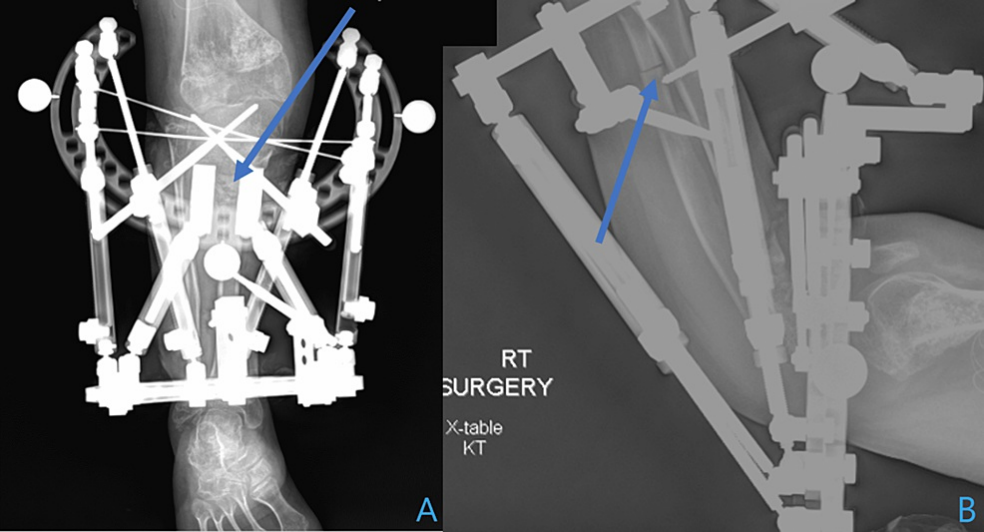
Immediate post-operative radiography Anteroposterior (A) and lateral (B) of the right leg immediately post-operatively demonstrating the circulator external fixator with a 155 5/8 ring proximally, a 105 full ring distally, six struts, and two z-plates used to offload the proximal struts. The proximal tibial and fibular osteotomy and distal fibular osteotomy fixation are pointed out (blue arrows). The external fixator is fixated to the proximal tibia and the distal tibia/fibula syndesmosis.

At this point, given the brace intolerance, the patient’s symptoms, and the severity of the deformity, surgical options were presented to the patient and her family. The patient and her family were informed that the deformity on the right could be explained largely via the proximal tibial recurvatum deformity, whereas the deformity on the left had about two-third of the deformity due to the tibia and one-third due to the distal femur. Given the preexisting diagnosis and the severity of the deformity, a circular external fixator would be used to allow the soft tissues to better tolerate the degree of correction. Having understood all of this, the patient and her family elected to proceed with a right tibia-fibular osteotomy with the application of a circular external fixator (Figures 2A, 2B, 3A, 3B, 4A, 4B).

**Figure 5:**
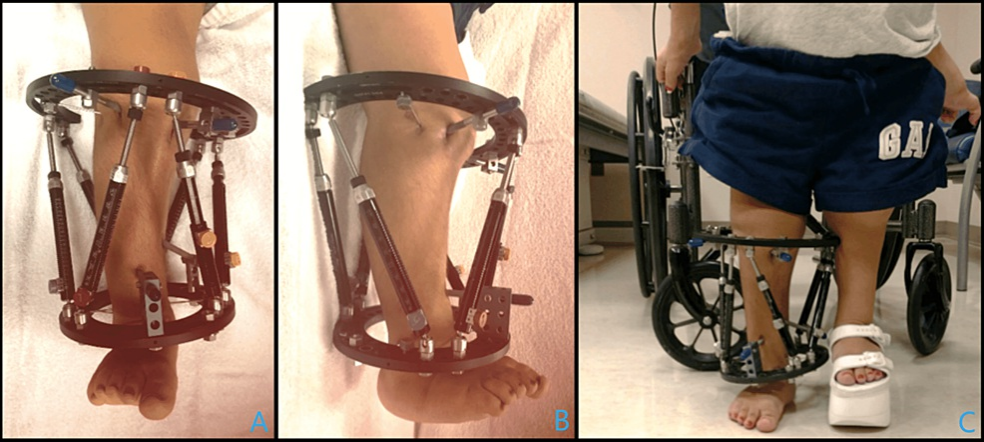
Eleven-week follow-up Clinical photographs at 11 weeks post-operatively demonstrate a stable construct (A, B) and good weight-bearing ability (C).

Correction and lengthening began one week postoperatively with a detailed external fixator adjustment protocol provided to the family. At four weeks postoperatively, the two z-plates were removed from struts 1 and 2 and medium struts were connected to the proximal ring. At eight weeks postoperatively, the Ilizarov 1.8 mm wire was removed from the proximal tibial ring. At this point, the patient was instructed to progress to weight-bearing as tolerated. At 11 weeks postoperatively, the patient had a stable construct and the ability to tolerate weight bearing (Figures 5A-5C).

**Figure 6:**
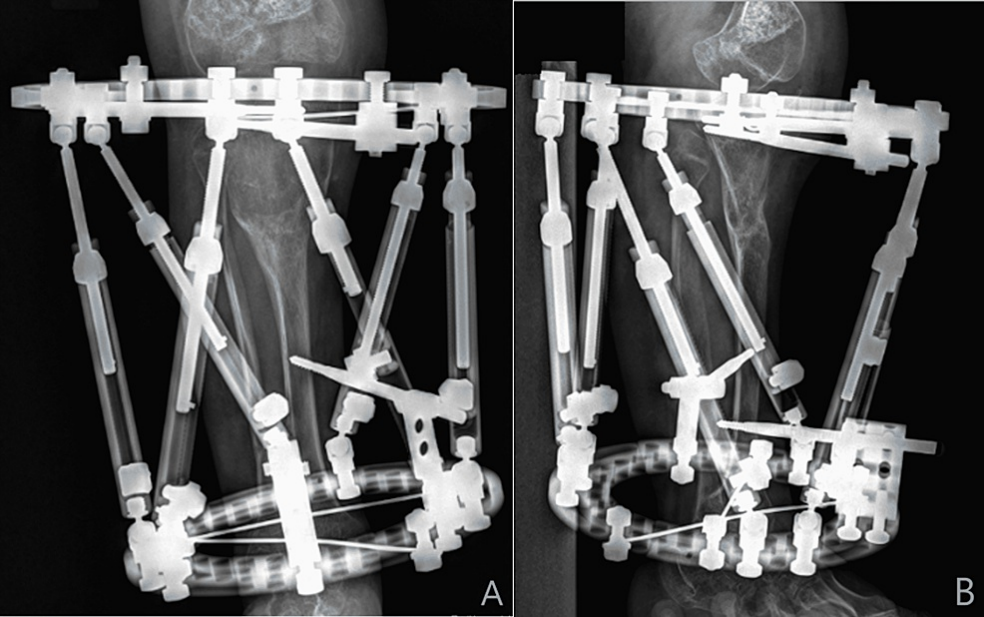
Radiography at five months postoperatively Anteroposterior (A) and lateral (B) radiographs of the leg five months post-operatively demonstrate a proximal healing tibial osteotomy site with callus and sclerosis.

Leg radiographs were ordered sequentially every month following surgery. At five months postoperatively, good bony healing of the proximal tibia and fibular osteotomy sites were seen (Figures 6A, 6B).

Given the radiographic evidence of good healing of the osteotomy sites and near-complete fusion of the distal femoral and proximal tibial physis, the decision to remove the external fixator was seen.

Follow-up

**Figure 7:**
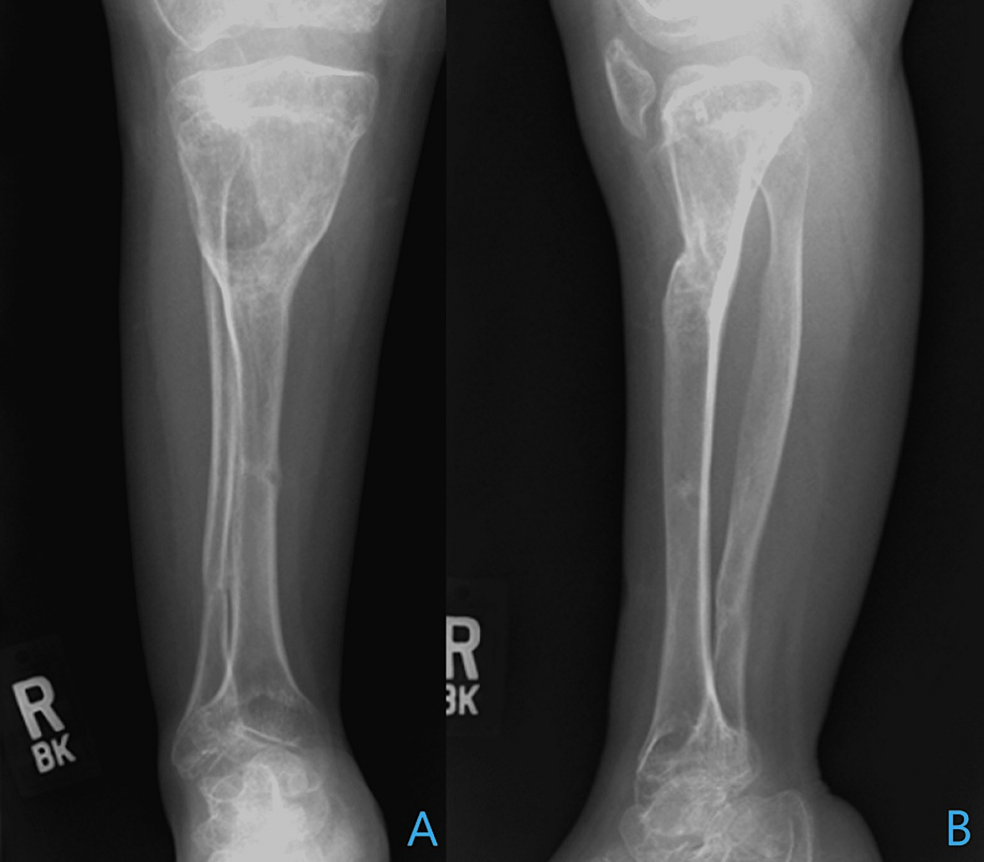
Radiography at one year follow-up Weight-bearing anteroposterior (A) and lateral (B) radiographs of the right tibia and fibula one year postoperatively demonstrate complete bony healing of the tibial and fibular osteotomy sites.

At the one-year follow-up, the patient was extremely pleased with the surgical outcome and reported good subjective stability. Physical examination revealed well-healed pin sites, a right knee range of motion from 0 to 100 degrees, and good alignment of the tibia. She was able to ambulate without external aid. Radiography revealed maintenance of the bony healing from the radiograph (Figures 7A, 7B) At this point, the discussion regarding surgical treatment of the contralateral limb was undertaken, which would not only address the contralateral deformity, but also the limb-length discrepancy.

## Discussion

Genu recurvatum is a reported though less common deformity in patients with spondyloepiphyseal dysplasia. Our case demonstrated good bony healing, an improvement in radiographic criteria [7], and excellent clinical outcome using a hexapod fixator with two z-plates.

The current treatment for symptomatic genu recurvatum in children having failed conservative management is the standard Ilizarov technique [7,13,14]. Babu et al. describe this technique for proximal tibial recurvatum deformity in a series of nine children (2 SEDC patients). They demonstrated a correction of the tibial recurvatum angle by 21 degrees and the tibial plateau tilt by 24.51 degrees. However, in their cases the average recurvatum angle was 28 degrees preoperatively. Our case with a recurvatum deformity of nearly 90 degrees rendered this solution significantly more difficult. Further, given the severity of the deformity, as well as anatomic and soft tissue constrains, open acute corrective osteotomies would have also been rendered difficult. Hence an alternative method that employed gradual correction would be a safe and accurate option.

The hexapod system served as an alternative that was more accurate, less labor intensive, yet facilitated a gradual correction through distraction osteogenesis using the same principles as the standard Ilizarov technique. Further, the use of the two z-plates allowed us to respect the functional limit of 30 degrees on the ring-strut angle. It has been shown that the ring-strut angle is a critical factor in the stability of a TSF [15]. Further, the standard TSF is not without its biomechanical limitations [16]. Considering these factors made the hexapod system with two z-plates a better choice considering the magnitude of this patient’s deformity.

Of note, in the conversation of long-term management of spondyloepiphyseal dysplasia is the concern for early osteoarthritis in these children [9,10]. The correct orthopedic intervention may unload the joint surfaces, increase function, and potentially reduce in the incidence of early arthritis in these children. In our case, the patient’s preoperative x-rays did show significant destruction of the knee joint. It is difficult to assess the post-operative joint space in the setting of the prior large sagittal plane deformity seen; however, articular irregularities and subchondral sclerosis can be seen. These are likely explained by several years of withstanding abnormal joint forces due to the extreme genu recurvatum deformity.

## Conclusions

This case demonstrates good correction to nearly 0 degrees of tibial recurvatum using a hexapod fixator with two z-plates. Adequate length struts should be chosen when constructing a TSF so that ring-strut angles of 30 degrees or less are avoided. The use of a standard TSF in atypical pediatric patients should be pursued sparingly and a hexapod fixator with the use of two Z-plates may help ensure that the ring strut angles of 30 or greater are avoided. In our patient, this device facilitated appropriate healing and a good clinical outcome. Larger scale studies are needed regarding the use of this technique in children with spondyloepiphyseal dysplasia.
